# Sea Cucumber Viscera Processed by Protease Hydrolysis Combined with *Cordyceps militaris* Fermentation Protect Caco-2 Cells against Oxidative Damage via Enhancing Antioxidant Capacity, Activating Nrf2/HO-1 Pathway and Improving Cell Metabolism

**DOI:** 10.3390/antiox13080988

**Published:** 2024-08-14

**Authors:** Rui Mi, Zhiyu Fu, Jingwei Jiang, Shan Gao, Xiaoyan Guan, Xuda Wang, Zunchun Zhou

**Affiliations:** Liaoning Ocean and Fisheries Science Research Institute, Liaoning Academy of Agricultural Sciences, Dalian 116024, China; lianyi7432@sina.com (R.M.); hkyfzy@126.com (Z.F.); weijingjiang@live.cn (J.J.); gs_7920@163.com (S.G.); guanxiaoyan201@163.com (X.G.); wangxuda860@sina.com (X.W.)

**Keywords:** fermented sea cucumber viscera protease hydrolysates (FSVHs), oxidative damage, *Cordyceps militaris*, antioxidant capacity, metabolomics, mechanism analysis

## Abstract

Excessive reactive oxygen species (ROS) may lead to oxidative damage and metabolic disorder. The pathogenesis of human bowel inflammation is closely related to oxidative damage of intestinal epithelial cells caused by ROS. This study aimed to explore the high-value utilization of the byproducts of sea cucumber in antioxidant food for colitis prevention. The technology of protease hydrolysis combined with *Cordyceps militaris* fermentation was used to obtain fermented sea cucumber viscera protease hydrolysates (FSVHs). The results revealed that FSVH could enhance antioxidant capacity and alleviate oxidative damage and apoptosis by activating the Nrf2/HO-1 pathway and triggering the self-protection immune mechanisms. Moreover, the FSVH supplementation could upregulate antioxidant-related metabolic pathways of Caco-2 cells such as glutathione metabolism, confirming the enhanced antioxidant capacity of damaged cells. In summary, FSVH could exert protective effects on Caco-2 cells in response to oxidative damage, providing a promising prospect for sea cucumber resource utilization and colitis prevention.

## 1. Introduction

Oxidative stress can cause an imbalance between oxidation and antioxidant action in vivo. Reactive oxygen species (ROS) are generated and accumulated in cells extensively [1]. Excessive production of ROS will lead to protein oxidation and DNA damage, further causing pathological changes such as inflammation, cancer and neurodegeneration in the body [2]. The intestinal epithelium plays an important role in absorbing nutrients and acts as a physical barrier between the host and the environment [3]. The pathogenesis of human bowel inflammation is closely related to oxidative damage of intestinal epithelial cells caused by ROS [4]. Diffuse inflammatory cell infiltration and abscess can induce the mass production of ROS, which may affect the permeability of the intestinal epithelial barrier, leading to pathogen invasion, inflammatory cell infiltration and excessive damage from inflammation [5,6]. In addition, excess ROS production may also reduce the activities of antioxidant enzymes in the injured cells. Many researchers have proposed antioxidant and ROS-targeted therapies to prevent or reduce colon inflammation [7]. Therefore, screening and studying antioxidants that can effectively reduce the colon cell damage caused by oxidative stress is one of the effective strategies for prevention and control of colitis and improving the health-related quality of patients [8,9].

Sea cucumber is a kind of marine resource with high edible and medicinal values. It is rich in a variety of bioactive substances, including proteins and peptides, polysaccharides, saponins, etc. [10,11,12]. Many researchers have found that these substances possess physiological activities such as antifatigue, immune regulation and antitumor [13,14,15]. The deep processing of edible varieties of sea cucumber (such as *Apostichopus japonicus*) is mainly aimed at the efficient application of the sea cucumber body wall, and many byproducts will be produced in the processing process, including sea cucumber viscera and boiling liquid [16]. The viscera of sea cucumber contain the digestive, respiratory, water pipe and reproductive systems after processing the body wall [17]. Studies have shown that these byproducts contain functional active substances similar to the body wall, most of which are discarded, causing a great waste of resources [18]. If these byproducts can be effectively and rationally used, they can improve the added values of sea cucumber application and alleviate the problems of resource waste and environmental pollution. Fermentation is an economic method for producing microbial biomass using byproducts as a nutritional food source, which is starting to be adopted in human foods with health promoting effects [19]. It is a metabolic process that could make use of many kinds of microorganisms, including fungi (such as *Cordyceps militaris*), which could convert protein into energy [20]. *C. militaris* is an entomogenous fungi that has been widely used as a tonic in China for hundreds of years [21]. *C. militaris* has been proved to possess protective effects against asthma and nephropathy [22,23]. At the same time, *C. militaris* is also shown to have antioxidant, anti-inflammation and anticancer activities [24,25]. H_2_O_2_ is an intermediate product of oxidative metabolism in vivo, which can cause oxidative stress damage to cells. Human colonic adenocarcinoma Caco-2 cells are typically used to investigate the mechanism of intestinal barrier damage occurrence and restoration. When the cells are overfilled, they exhibit characteristic differentiation of intestinal epithelial cells and have a similar metabolic enzyme system and secretion functions as intestinal epithelial cells [26]. In the previous study, we studied the antioxidant effect of sea cucumber viscera enzymatic hydrolysates (SVHs) [18]. However, there are a limited number of articles addressing the applications of sea cucumber viscera as of yet, and this study would be innovative and necessary.

Thus, in this study, the fermented sea cucumber viscera enzymatic hydrolysates (FSVHs) were obtained by treating sea cucumber viscera through protease hydrolysis combined with *C. militaris* fermentation technology. Further, the H_2_O_2_-induced oxidative damage model of Caco-2 cells was established to analyze the antioxidant effect and explore the protective effect of FSVH on the inhibitory mechanism of apoptosis induced by oxidative stress. In addition, metabolomics was used to evaluate the positive effects of FSVH on the related metabolic pathways, so as to provide a theoretical reference for the development and utilization of sea cucumber viscera as nutritious and functional foods.

## 2. Materials and Methods

### 2.1. Preparation of the Experimental Samples and Component Detection

The fermented sea cucumber viscera enzymatic hydrolysate (FSVH) samples were obtained by means of protease hydrolysis, autoclaving, adding *C. militaris* liquid strains and further fermentation. Sea cucumber viscera enzymatic hydrolysate (SVH) samples were obtained via protease hydrolysis and autoclaving. Specifically, the viscera of sea cucumbers were soaked in distilled water for 8 h, and this was repeated 3 times to remove salt. Distilled water was added for homogenization, and the papain with an activity unit of 10^5^ U/g was added. The temperature was controlled at 50 °C, and enzymatic hydrolysis was carried out with magnetic stirring for 2 h. After filtration and impurity removal, SVH was obtained by sterilizing at 115 °C for 30 min. Then, the activated *C. militaris* liquid strain was inoculated into sea cucumber viscera enzymatic hydrolysates, aerated and stirred for 7 days at 22 °C. After that, the fermentation broth was sterilized at 115 °C for 30 min, and the supernatant was obtained via centrifugation and freeze-dried into powder to obtain FSVH.

The contents of protein and peptide were determined using Kjeldahl nitrogen determination. The content of polysaccharide was determined via the colorimetric method. Determination of saponin and cordycepin was conducted using high-performance liquid chromatography (HPLC).

### 2.2. Cell Culture and Treatments

The human colon tumor Caco-2 cell line was provided by the American Tissue Culture Collection (ATCC, Rockville, MD, USA). The cells were cultured in EMEM medium with 20% fetal bovine serum (FBS). After incubation with samples, the effects of SVH and FSVH samples at different concentrations (0.016 mg/mL, 0.08 mg/mL, 0.4 mg/mL, 2 mg/mL, 10 mg/mL and 50 mg/mL) on cells were detected, the appropriate sample concentration was selected to treat the cells for subsequent tests. The cell models were established with 600 μ mol/L H_2_O_2_ induced for 4 h [27]. After that, the cells were cultured in a 96-pore plate for 24 h incubation with samples. Then, the cells were split into three treatments, namely the control group (cells without treatment), model group (cells treated with H_2_O_2_) and intervention groups (cells intervened on with SVHs and FSVHs after treatment with H_2_O_2_). In addition, the intervention groups were divided into the 0.016 mg/mL group, 0.08 mg/mL group and 0.4 mg/mL group, respectively. After treatment, the viability of Caco-2 cells was determined using a CCK-8 kit.

### 2.3. Effect of Samples on Cell Growth Cycle and Apoptosis

After intervention, the cells were digested and fixed with ethanol at 4 °C for 12 h. The supernatant was then removed via centrifugation, and 500 μL of RNase A (50 μg/mL) were added to digest the RNA. Afterwards, the cells were stained at 4 °C with propidium iodide (PI) dye (50 μg/mL) for 30 min. Cells were then resuspended and mixed with fluorescent dyes Annexin V-FITC and PI at a volume of 1:1 for determining the degradation and apoptosis.

### 2.4. Measurement of Cells’ Membrane Potential

Detection was performed using the principle that JC-1 dye accumulates in mitochondria in a potential-dependent manner. After digesting and collecting the cells, 1 mL of JC-1 staining solution was added. After incubation at 37 °C for 20 min, the supernatant was aspirated and washed three times. Then, 2 mL of cell samples were mixed thoroughly, and the Caco-2 cells were examined with a fluorescence microscope. When the green fluorescence of the JC-1 monomer was detected, the excitation wavelength was 490 nm, and the emission wavelength was 530 nm. When the red fluorescence of the JC-1 polymer was detected, the excitation wavelength was 525 nm, and the emission wavelength was 590 nm.

### 2.5. Measurement of ROS and Related Enzyme Levels in Cells

ROS detection kits were provided by the Beyotime Bioengineering Institute in Shanghai, China. Briefly, cells were washed with PBS and incubated with DCFH-DA (10 μM) at 37 °C for 20 min. The fluorescence was detected using the Zeiss laser scanning confocal microscope (Carl Zeiss Microscopy, Jena, Germany) with an excitation wavelength of 488 nm and an emission wavelength of 525 nm. Malondialdehyde (MDA), catalase (CAT), superoxide dismutase (SOD)the name of the manufacturer, city, and country from where the equipment was sourced. and total antioxidant capacity (T-AOC) levels were evaluated and analyzed using enzyme-linked kits purchased from Nanjing Jiancheng Bioengineering Institute. The experiments were performed according to the instructions. Three parallel tests were set for each treatment to minimize errors.

### 2.6. Real-Time Quantitative PCR (RT-qPCR) Analysis

After incubation, the mRNA expression levels of heme oxygenase (HO-1) and quinone oxidoreductase 1 (NQO-1) were measured with fluorescence quantitative PCR. The primers used in this study are listed in Table 1. Total RNA was isolated and reversely transcribed into cDNA using a SYBR PrimerScrip RT-PCR kit (TaKaRa). The *q*PCR reaction mixture contained 10 μL of 2 × SYBR Green Mix, 2 μL of the cDNA, 0.4 μL of each primer (10 mM), 0.4 μL of 50 × ROX Reference Dye I and 6.8 μL of RNase-free water. The *q*PCR was conducted using an ABI7300 PCR detection system under the following conditions: initial denaturation at 95 °C for 30 s, followed by 40 cycles of 95 °C for 5 s and 60 °C for 30 s. Each sample was run in triplicates. The data were normalized with the 2^−△△Ct^ method.

### 2.7. Western Blot Analysis

The cells were collected according to the experimental groups and washed twice with pre-cooled PBS. Then, 1 mL of radio-immunoprecipitation assay (RIPA) buffer was added to the cell samples for lysis for 30 min, followed by centrifugation at 12,000 rpm for 5 min at 4 °C. The supernatant, which was the total cell protein, was absorbed into EP tubes. Nuclear proteins and cytoplasmic proteins were extracted using commercial kits (Beyotime, Shanghai, China). The protein content was tested using a BCA protein assay kit. Proteins were separated with 12% SDS-PAGE and transferred to a polyvinylidene fluoride membrane for Western blot analysis. The membrane was exposed to antibodies against the nuclear transcription factor erythroid 2-related factor 2 (NRF2), HO-1 and NQO-1. The protein signal was improved using horseradish peroxidase (HRP)-conjugated secondary antibody and was detected with a Tanon 6600 Multi fluorescence image analysis system (Tanon, Shanghai, China).

### 2.8. Untargeted Metabolomics Analysis

Cells were taken and lysed with 100 μL ultra-pure water and 300 μL methanol solution. Shake extraction for 5 min, centrifuge at 18,000 rpm for 10 min, transfer 300 μL of supernatant to EP tube and then use a vacuum drying instrument. The ultrapure water was redissolved with methanol (1:1) 100 μL, centrifuged at 18,000 rpm for 10 min, and 80 μL supernatant was taken and 5 μL was injected. HPLC-MS was used, and the metabolite was fractionated using a C18 column for metabolomics analysis. Turbo V electrospray ionization (ESI) was used for scanning analysis for quality detection. The atomizer and auxiliary gas were held by nitrogen. TOF MS showed a scanning range of *m*/*z* 50–1200.

### 2.9. Statistical Analysis

The data analysis was conducted with SPSS software v. 21.0, and the levels of significance were expressed as *p* < 0.05. The experiments were carried out in triplicate, and Duncan’s multiple range tests were used to assess the mean differences of mentioned parameters among different treatments. The LC-MS/MS data were analyzed using multivariate statistical analysis (PCA, PLSDA and OPLSDA), mining and identification of differential metabolites and pathway attribution (KEGG database).

## 3. Results

### 3.1. The Contents of SVH and FSVH

Table 2 shows the changes in the main components in FSVH. Compared with SVH, the protein contents of FSVH decreased to 32.35 g/100 g, the polysaccharide contents significantly increased to 19.53 g/100 g compared with SVH (*p* < 0.05) and the changes in saponin contents did not reach significant levels between the SVH and FSVH (*p* > 0.05). In addition, the cordycepin contents were probably 852.19 mg/kg in FSVH (*p* < 0.05), which were not detected in SVH. The contents of polysaccharides and cordycepin in FSVH increased significantly, while the contents of proteins and peptides decreased. This indicated that *C. militaris* could fully utilize protein as the nitrogen source through the fermentation process and effectively increase the contents of active substances.

### 3.2. Cell Viability and Oxidative Damage Model

#### 3.2.1. Cell Viability

**Figure 1:**
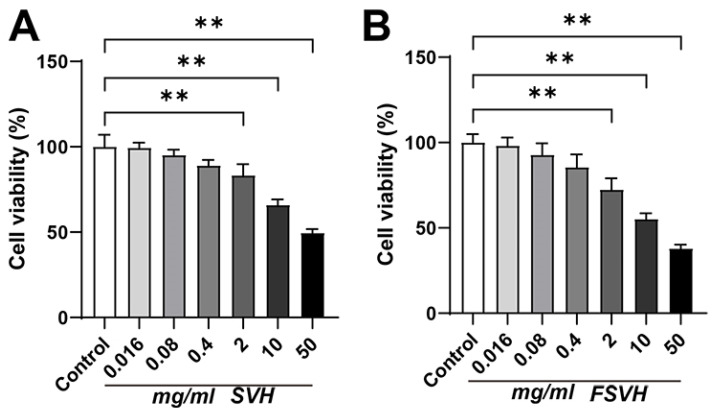
Effects of SVH (**A**) and FSVH (**B**) on cell viability of Caco-2 cells. Results were mean ± SD for three experiments. ** *p* < 0.01.

The effects of SVH and FSVH on cell viability are displayed in Figure 1. The activity of Caco-2 cells measured via the CCK-8 method decreased with increasing concentration in both the SVH and FSVH groups compared to the control group. For subsequent experiments, the two groups were selected at concentrations of 0.4 mg/mL, 0.08 mg/mL and 0.016 mg/mL. Cells did not exhibit inhibitory viability or growth promotion when exposed to SVH and FSVH concentrations that were found to be relatively safe for cellular events.

#### 3.2.2. Establishment of Oxidative Damage Model

**Figure 2:**
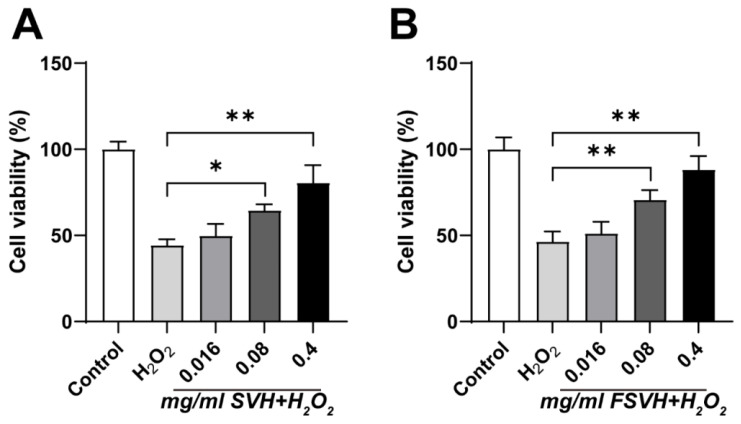
Protective effects of SVH (**A**) and FSVH (**B**) on H_2_O_2_-induced oxidative damage of Caco-2 cells. Results were mean ± SD for three experiments. * *p* < 0.05, ** *p* < 0.01.

Based on methods in the literature, the treatment conditions for inducing oxidative damage were an intervention time of 4 h and an H_2_O_2_ concentration of 600 μmol/L [27]. As depicted in Figure 2, the cell viability of the H_2_O_2_ group exhibited a significant decrease compared to the control group. Conversely, Caco-2 cell activity was observed to increase in both the SVH and FSVH groups at varying concentrations compared to the H_2_O_2_ group. The most protective effects were observed at concentrations of 0.4 mg/mL for both SVH and FSVH, which were subsequently selected for further experimentation.

### 3.3. Effects of SVH and FSVH on Oxidative Damage

#### 3.3.1. Cell Cycle

**Figure 3:**
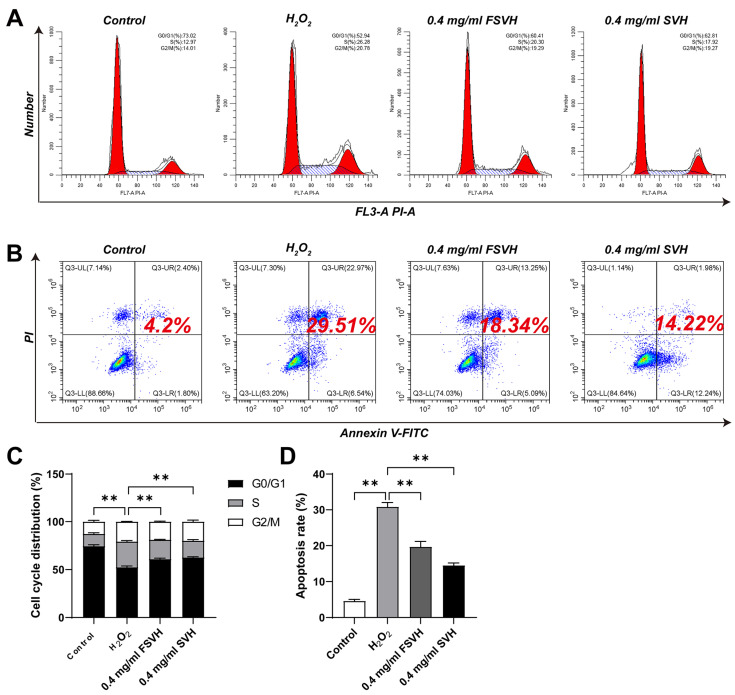
Effects of SVH and FSVH on cell cycle and apoptosis in H_2_O_2_-treated Caco-2 cells. Cell cycle analysis of Caco-2 cells (**A**,**C**). The apoptosis ratio of Caco-2 cells (**B**,**D**). Results were mean ± SD for three experiments. ** *p* < 0.01.

The effects of SVH and FSVH on the cell cycle are illustrated in Figure 3A,C. H_2_O_2_ inhibited the proliferation of Caco-2 cells by causing cell cycle arrest at the S phase. This oxidative stress-induced DNA fragmentation ultimately lead to cellular apoptosis and death. The group treated with 600 μM H_2_O_2_ increased the cells amounts at the S phase (26.28%) more markedly than the control group (12.97%), and the values reduced from 73.02% to 52.94% at the G1 phase. However, the ability of SVH and FSVH to attenuate cell cycle arrest was significant. Treatment with 0.4 mg/mL of SVH and FSVH resulted in a decrease in the cell counts at the S phase (20.30% and 17.92%, respectively) compared to H_2_O_2_ treatment (26.28%), while increasing the cells at the G1 phase from 52.94% to 60.41% and 62.81%. Based on the data analysis, the effect of FSVH was better than SVH, but there was no significant difference between the two intervention groups (*p* > 0.01).

#### 3.3.2. Cell Apoptosis

Apoptosis and the death of cells can be directly observed via flow cytometry in Figure 3B, the apoptotic cells in the H_2_O_2_-treated group (29.51%) were seven-fold higher than the control group (4.2%), suggesting that H_2_O_2_ could dramatically prompt the apoptosis of Caco-2 cells under oxidative damage conditions, increasing the rate of late apoptosis and cell death. The intervention of FSVH and SVH could remarkably reduce the apoptotic rates of Caco-2 cells (*p* < 0.01); FSVH could act on the late apoptosis of cells and decrease cell death obviously. The effects of SVH and FSVH on cell apoptosis are shown in Figure 3D; the apoptosis rate of Caco-2 cells treated with 0.4 mg/mL FSVH decreased from 29.51 to 14.22%, which was lower than the 0.4 mg/mL SVH treatment (18.34%). However, there was no significant difference between the two intervention groups (*p* > 0.01).

#### 3.3.3. Cells’ Membrane Potential

**Figure 4:**
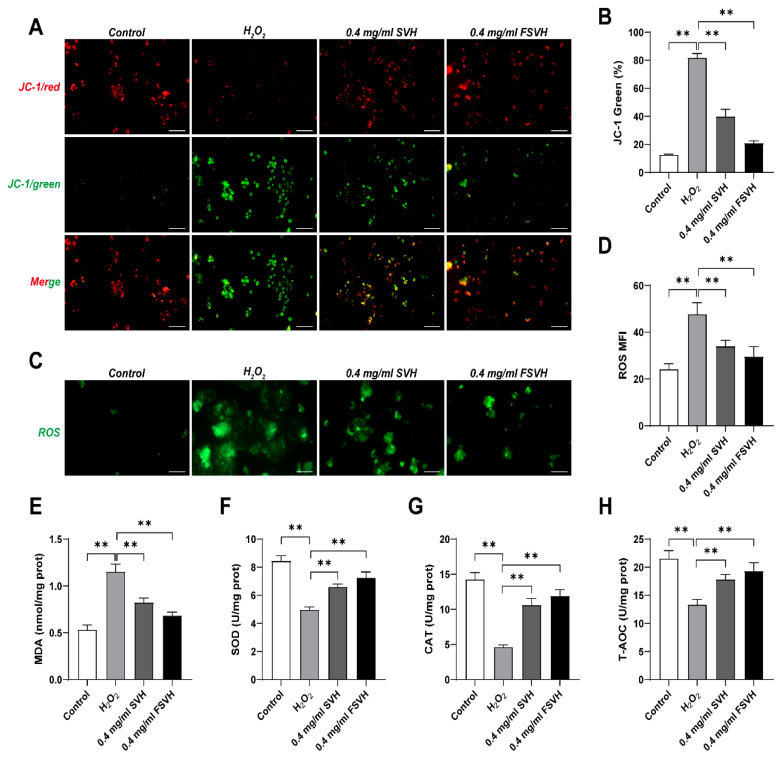
Effects of SVH and FSVH on the mitochondrial membrane potential, the ROS levels and the antioxidant enzyme activities in H_2_O_2_-treated Caco-2 cells. The mitochondrial membrane potential of Caco-2 cells (**A**,**B**). The ROS production in Caco-2 cells (**C**,**D**). The levels of MDA (**E**). The levels of SOD (**F**). The levels of CAT (**G**). The levels of T-AOC (**H**). Results were mean ± SD for three experiments. ** *p* < 0.01.

The mitochondrial membrane potential is usually reduced under oxidative damage conditions. In normal mitochondria, JC-1 accumulates in the mitochondrial matrix to form a polymer, which emits red fluorescence. When the mitochondrial membrane potential decreases, JC-1 can only exist in the cytoplasm as a monomer, producing green fluorescence. Therefore, the increase in JC-1 green generally indicates a decrease in mitochondrial membrane potential. The effects of SVH and FSVH on the mitochondrial membrane potential are displayed in Figure 4A. The cells after FSVH intervention were covered with distinct red fluorescence, while the fluorescence arrangement was messy and weak in SVH treatment, indicating that FSVH could better reduce the production of reactive oxygen species, enhance the vitality of damaged mitochondria and repair the cells to normal than SVH. In Figure 4B, compared with the model group, the proportion of JC-1 green in the intervention groups decreased obviously (*p* < 0.01); the proportion of JC-1 green in SVH intervention group (39.94%) was higher than that in the FSVH group (20.83%), which indicated that mitochondrial membrane potential was increased, so the mitochondrial viability in FSVH treatment was higher than in the SVH treatment.

#### 3.3.4. The ROS Level

The intracellular ROS levels were measured under the condition of oxidative damage, Figure 4C,D shows that H_2_O_2_-induced toxicity increased the ROS level. The ROS level is represented by mean fluorescence intensity (MFI). MFI in cells treated with H_2_O_2_ increased to 47.71 compared with untreated cells. Both the SVH and FSVH treatments could decrease the level of ROS significantly (*p* < 0.01), and the MFI was 33.98 and 29.52, respectively. Notably, FSVH at 0.4 mg/mL reduced the intracellular ROS level lower than that obtained using SVH, but there was no obvious difference between FSVH and SVH (*p* > 0.01).

#### 3.3.5. Cytoprotective Effect

The effects of SVH and FSVH on the antioxidation of cells are displayed in Figure 4E–H. The H_2_O_2_ group exhibited an obviously higher level of MDA activity than the control group (116.25%, *p* < 0.01). Comparatively, FSVH could remarkably reduce the contents of MDA (*p* < 0.01) (Figure 4E). The activity of SOD decreased 41.06% in the H_2_O_2_ group compared with the control group. The activities of SOD in the SVH and FSVH groups increased by 32.69% and 45.81% compared with the H_2_O_2_ group, respectively (Figure 4F). The control group showed the highest CAT activity among treatments, while the lowest value presented in the H_2_O_2_ group (*p* < 0.01). SVH and FSVH could both signally improve the CAT levels in damaged cells, and the cytoprotective effect was more obvious in the FSVH group (*p* < 0.01) (Figure 4G). T-AOC levels were increased via SVH and FSVH intervention, exhibiting levels 33.47% and 44.66% higher than the H_2_O_2_ group, respectively (Figure 4H). Overall, compared with SVH, FSVH improved cell activity and led to the avoidance of early cell apoptosis by enhancing mitochondrial activity, antioxidant activity and ROS scavenging more effectively.

### 3.4. FSVH Promoted Antioxidant Defense via Activation of the Nrf2 Signaling Pathway

**Figure 5:**
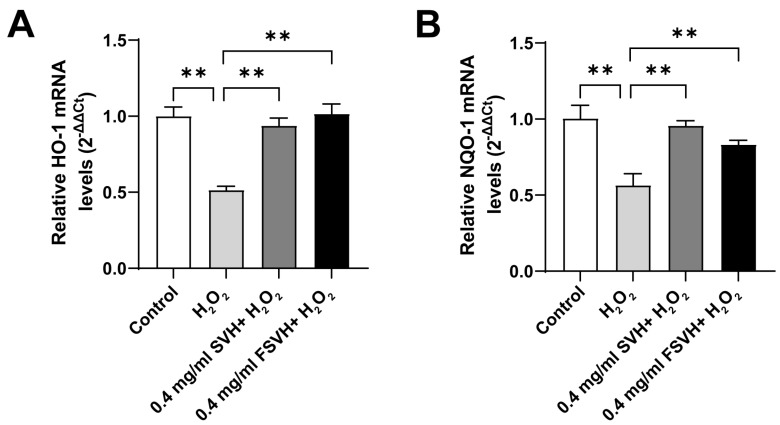
Effects of SVH and FSVH on the mRNA expression levels of HO−1 and NQO−1 in H_2_O_2_-treated Caco-2 cells. The mRNA levels of HO−1 (**A**) and NQO−1 (**B**). Results were mean ± SD for three experiments. ** *p* < 0.01.

The Nrf2 signaling pathway is the most important endogenous antioxidant stress pathway. In order to further study the intrinsic antioxidant mechanism of the samples, the expression of related genes in the Nrf2 pathway was analyzed. As shown in Figure 5A,B, mRNA expression of HO-1 and NQO-1 in Caco-2 cells in the H_2_O_2_ group decreased compared with the control group. Compared with the H_2_O_2_ group, the expression of HO-1 mRNA and NQO-1 mRNA in Caco-2 cells significantly increased in the SVH and FSVH groups at 0.4 mg/mL concentrations (*p* < 0.01). The difference between 0.4 mg/mL SVH and 0.4 mg/mL FSVH was not obvious (*p* > 0.01). Meanwhile, the mRNA expression of HO-1 was upregulated higher with FSVH intervention compared with SVH, but SVH had more effects on the upregulation of NQO-1 mRNA expression. These results indicated that FSVH and SVH could activate the expression of genes in the antioxidant pathway, which might better elucidate the antioxidant mechanism of the samples.

**Figure 6:**
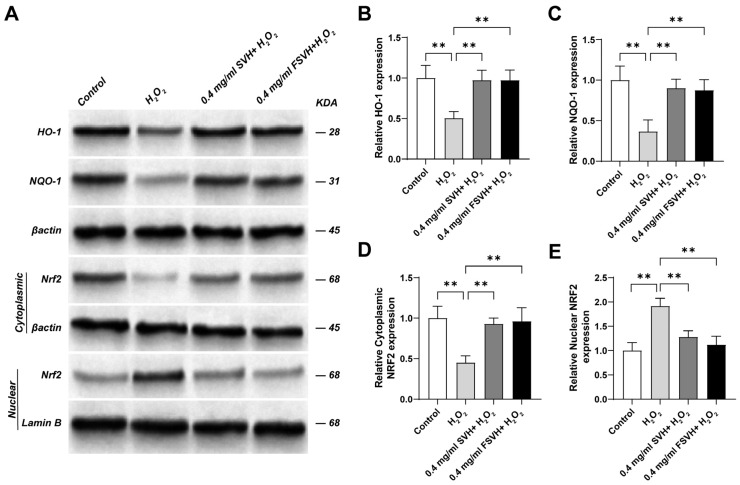
Effects of SVH and FSVH on the protein expression levels of HO−1, NQO−1 and NRF2 in H_2_O_2_-treated Caco-2 cells. The protein expression levels of HO−1 (**A**,**B**), NQO−1 (**A**,**C**), cytoplasmic NRF2 (**A**,**D**) and nuclear NRF2 (**A**,**E**). Results were mean ± SD for three individual experiments. ** *p* < 0.01.

Further Western blot analysis was performed to analyze the expression of antioxidation pathway-related proteins. As shown in Figure 6A, the protein expressions of HO-1, NQO-1 and NRF2 in the cell cytoplasm of Caco-2 in the H_2_O_2_ group were decreased, while the protein expression of NRF2 in the nucleus was increased. Figure 6B–D present a quantitative analysis of the Western blot results. Compared to the H_2_O_2_ group, the protein expressions of HO-1, NQO-1 and NRF2 in the cell cytoplasm of Caco-2 in the SVH and FSVH groups were increased (*p* < 0.01), while the protein expressions of NRF2 in the nucleus were decreased (*p* < 0.01) (Figure 6E). The effect of FSVH was better than that of SVH, but the difference between 0.4 mg/mL SVH and 0.4 mg/mL FSVH was not obvious (*p* > 0.01). Thus, the significant changes in the expression of proteins related to the antioxidant pathway not only indicated the protective effects of FSVH and SVH on cells, but also indicated the mechanism of their protective effects by activating the antioxidant pathway, which was consistent with the mRNA expression.

### 3.5. Variations in the Cells’ Metabolome

**Figure 7:**
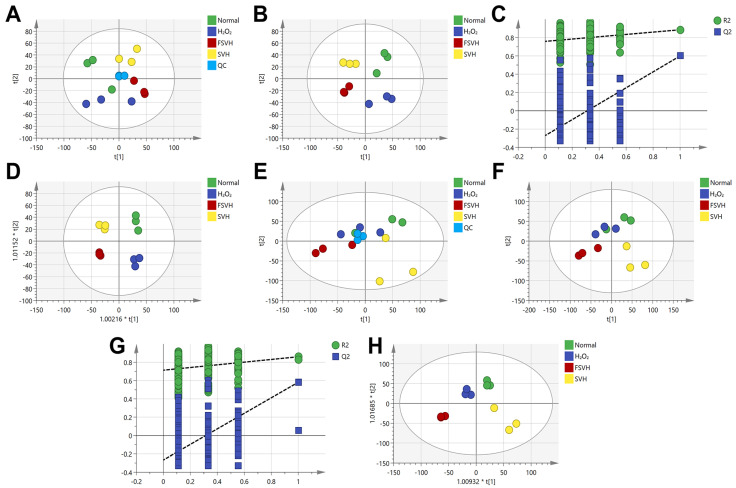
The PCA, PLS-DA, replacement verification plot and OPLS-DA of the control, H_2_O_2_, SVH and FSVH groups. (**A**) PCA score plot in negative ion mode (R^2^X = 0.492, Q^2^ = 0.0639). (**B**) PLS-DA score plot in negative ion mode (R^2^X = 0.528, R^2^Y = 0.919, Q^2^ = 0.721). (**C**) Replacement verification plot in negative ion mode; intercept of the regression Q^2^ = −0.271272. (**D**) OPLS-DA score graph in negative ion mode. (**E**) PCA score plot in positive ion mode (R^2^X = 0.508, Q^2^ = 0.0668). (**F**) PLS-DA score plot in positive ion mode (R^2^X = 0.552, R^2^Y = 0.884, Q^2^ = 0.632). (**G**) Replacement verification plot in positive ion mode; intercept of the regression Q^2^ = −0.26698. (**H**) OPLS-DA score graph in positive ion mode.

The metabolomics experiments were conducted to elucidate the protective mechanism of FSVH. Respectively, 294 and 637 metabolites were obtained in the negative and positive ion modes. Principal components analysis (PCA) was used to analyze the differences between groups (Figure 7A,E). An obvious clustering was observed in the negative ion mode. In order to further investigate the differences, the data were examined using PLS-DA (Figure 7B,F) and OPLS-DA (Figure 7D,H), and permutation analysis was performed for reliability confirmation (Figure 7C,G). The results indicated that four groups were more decentralized in the negative ion mode, and the samples in the group were centralized (Figure 7). Comparatively, the quality control group showed a better clustering in the negative ion mode. The metabolomic profiles of the Normal (control) and H_2_O_2_ (model) groups and the two sample groups were independently distributed, suggesting that the metabolite profile of the cell was altered after FSVH intervention.

**Figure 8:**
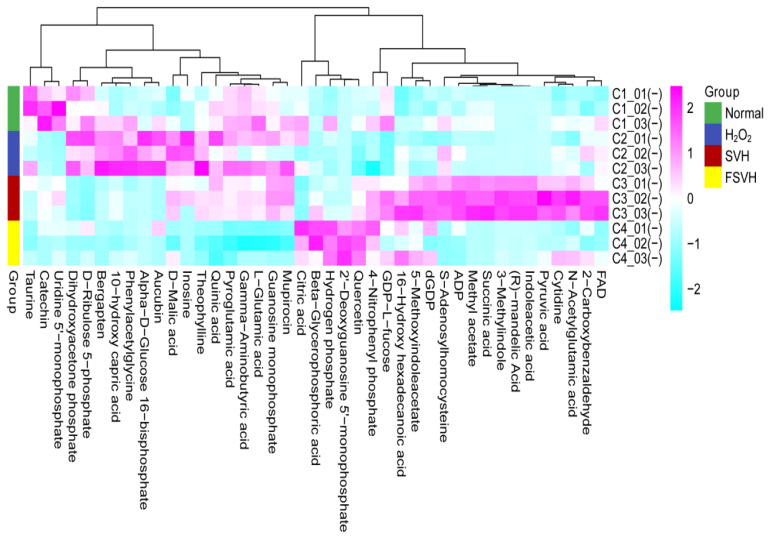
Heat map of differential metabolites in negative ion mode.

Differential metabolites with groups that matched with VIP (variable importance in the projection) > 1.0, FC (fold change) > 1.5 or FC < 0.67 and *p* < 0.05 were screened. As shown in Table 3, there were 49 metabolites in the negative mode. Moreover, 26 metabolites in the negative mode were signally regulated in the H_2_O_2_ group compared to the normal group. After SVH and FSVH intervention, 36 and 41 metabolites in the negative mode were modulated compared to the H_2_O_2_ group, respectively. These differential metabolites in the four groups were independently distributed in PCA and PLS-DA analysis (Figure 7A–D). Therein, 13 metabolites that were upregulated in the H_2_O_2_ group were downregulated with FSVH. In addition, 4 metabolites were downregulated in the H_2_O_2_ group that were upregulated with FSVH, as shown in the heat map in Figure 8. Fold change of the differential metabolites in each comparison group is shown in Table 4. Differential metabolites like 2′-Deoxyguanosine 5′-monophosphate and inosine were up- or downregulated significantly compared to the H_2_O_2_ vs. Normal group.

**Figure 9:**
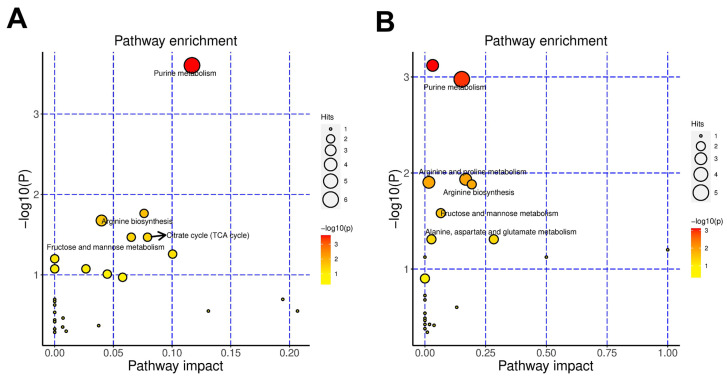
KEGG pathway analysis of SVH (**A**) and FSVH (**B**) groups. Each dot indicates one metabolic pathway; the size and shade of points represent the correlation with the influence of the metabolic pathway.

The top ten enriched metabolic pathways in each group were purine metabolism; arginine biosynthesis; tryptophan metabolism; fructose and mannose metabolism; citrate cycle (TCA cycle); glycolysis/gluconeogenesis; alanine, aspartate and glutamate metabolism; cysteine and methionine metabolism; arginine and proline metabolism and nicotinate and nicotinamide metabolism in the SVH group (Figure 9A) and butanoate metabolism; purine metabolism; arginine and proline metabolism; pyrimidine metabolism; arginine biosynthesis; fructose and mannose metabolism; glutathione metabolism; alanine, aspartate and glutamate metabolism; linoleic acid metabolism and D-Glutamine and D-glutamate metabolism in the FSVH group (Figure 9B). Among them, purine metabolism; arginine biosynthesis; fructose and mannose metabolism and alanine, aspartate and glutamate metabolism were enriched in the two groups simultaneously. In addition, glucose metabolism and amino acid metabolism were triggered in the SVH group. Arginine and proline metabolism and glutathione metabolism had the higher contribution value in the FSVH group, which had a crucial influence on cellular protection.

## 4. Discussion

Fermentation can be used to produce more active ingredients, enhance nutritional value, improve flavor and extend shelf life, improving the original substance composition of seafood [28]. A new form of sea cucumber product was developed via protease hydrolysis combined with *C. militaris* fermentation. The antioxidant activity and mechanism of the oxidative damage model of Caco-2 cells induced with H_2_O_2_ were analyzed, which provided the basis for product function development.

In the present study, SVH and FSVH both could improve cell viability, resume normal growth cycle, inhibit cell apoptosis and increase mitochondrial membrane potential, suggesting that the sea cucumber viscera protease hydrolysates could exert cellular protective effects and reduce oxidative damage of Caco-2 cells effectively. In addition, it was worth noting that the protective benefits were more obvious in the FSVH compared with the SVH group. This could be that the contents of functional components presented in FSVH, mainly including polysaccharides and cordycepin, increased significantly after fermentation treatment combined with *C. militaris*. The polysaccharides could inhibit cell apoptosis, improve the immune response and enhance antioxidant capacity by preventing DNA damage, inhibiting apoptotic gene expression, activating macrophages, suppressing viral reproduction and inducing cellular activity factors [29]. Moreover, the cordycepin with protein kinases’ inhibitive activity would also play positive roles and create the synergistic effects with the polysaccharides in promoting cell regeneration processes, reducing inflammatory reactions and reducing toxin accumulation [30]. Previous studies also showed that fermentation with *C. militaris* could significantly improve the bioactivity of soy whey by improving the ability of nerve cells to resist oxidative damage [21]. It could be seen that fermentation with *C. militaris* might be an effective way to process byproducts and enable more active substance synthesis.

After the oxidative damage induced with H_2_O_2_, Caco-2 cells in the model group induced the changes in the cell cycle and apoptosis processes, all of which were related to the activation of ROS via oxidative damage. ROS accumulation can destroy organelles and cause growth retardation and metabolism disorders [31,32]. Mitochondria are the main source of ROS. Excessive superoxide anion (O^−2^) in ROS free radicals can cause lipid peroxidation and sulfhydryl oxidation, damage the mitochondrial membrane and cause mitochondrial cleavage [33]. Previous studies have shown that fermentation with *C. militaris* increased the bioactivity of *Haliotis discus hannai*, which could prospectively be used as a functional food processing method with enhanced anti-inflammation and ROS inhibition effects [20]. Similarly, in the present study, supplementation with FSVH restored A-TOC, SOD, CAT and MDA levels in vivo, indicating that FSVH could reduce H_2_O_2_-induced oxidative damage. This might be due to the sea cucumber polysaccharides with sulfated hydroxyl groups containing large amounts of chondroitin sulfates, which might effectively enhance the phagocytic activity of cells and eliminate radicals [34]. After fermentation treatment, the polysaccharide molecules were improved with glycosidic bond dissociation and energy-releasing reactions, which could also possibly be linked with the enhancement of the antioxidant capacity. In addition, the conversion and synthesis of cordycepin after *C. militaris* fermentation could scavenge free radicals, alleviate peroxidative injury and improve oxygen-carrying capacity of cells. Moreover, the cordycepin could produce hydrogen gas, deactivate aminotransferase and reduce tissue damage, contributing to a higher antioxidation capacity indirectly [35]. MDA is a small molecule product formed during the metabolism of lipid peroxides. MDA content can be used to measure the degree of lipid peroxidation [36]. The decrease in MDA level also indicated that FSVH supplementation could maintain stable and versatile membrane properties and protect cells against exogenous oxidative damage.

**Figure 10:**
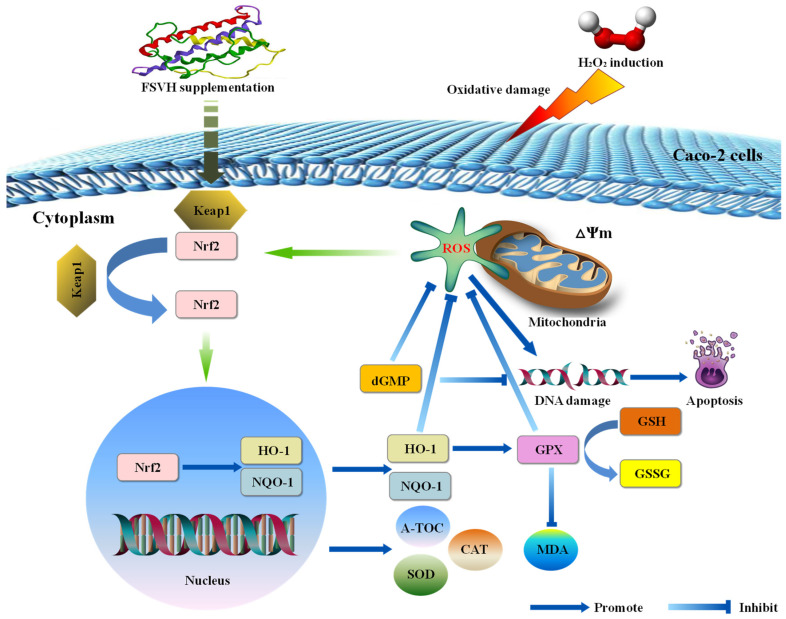
Schematic diagram of protective mechanism of FSVH supplementation against H_2_O_2_-induced oxidative damage of Caco-2 cells.

In the analysis of antioxidant mechanisms, the Nrf2/HO-1 pathway plays a vital role in the cellular antioxidant defense system [37]. Kelch-like ECH-associated protein 1 (Keap1) is the major repressor protein of nuclear factor erythroid 2-related factor 2 (Nrf2), which could activate the transcription of antioxidants and detoxify related genes [38,39]. Generally, Keap1 can interact with Nrf2 to promote the ubiquitination of Nrf2 protein, leading to the degradation of Nrf2 [40]. Under oxidative stress, ROS can modify the cysteine residues of the Keap1 protein for Nrf2 production, inducing the transcription of a series of antioxidative and cytoprotective genes, including HO-1 and NQO-1 [41]. As a transcription factor, Nrf2 is mainly involved in post-transcriptional regulation of ubiquitination, so the gene expression level had not been analyzed in this study. A schematic diagram of the protective mechanism of FSVH supplementation against H_2_O_2_-induced oxidative damage of Caco-2 cells is displayed in Figure 10. FSVH could significantly decrease Nrf2 in the nucleus but increase Nrf2 in the cytoplasm and increase HO-1 and NQO-1 levels. It could be that the Nrf2-Keap1 complex was dispersed which stimulated Nrf2 to transfer into the nucleus and transcriptionally activate the downstream target genes HO-1 and NQO-1. Then, the Nrf2/HO-1 pathway of Caco-2 cells was activated as a result of H_2_O_2_-induced oxidative damage, implying that FSVH supplementation could effectively improve the protein synthesis and gene expression involved in signaling pathways and exert an antioxidative action on Nrf2/HO-1 pathway regulation. Previous studies showed that sea cucumber peptides could improve exercise performance and express an antifatigue effect by activating the Nrf2 signaling pathways in mice [13]. Cordycepin also could regulate the oxidative stress pathway as elucidated with transcriptome analysis, including activating Nrf2, NQO-1 regulation, exerting an antioxidative effect and improving intestinal inflammation under oxidative stress [42]. Therefore, FSVH could counteract the oxidative damage induced with H_2_O_2_ via activating the Nrf2/HO-1 pathway.

As for the metabolomics analysis in this study, it was found that 2′-Deoxyguanosine 5′-monophosphate (dGMP) was upregulated with higher abundance in the FSVH group compared with the oxidative damage group through the analysis of differential metabolites. Nucleotides are the precursors of synthetic biological macromolecular ribonucleic acid (RNA) and deoxyribonucleic acid (DNA). There are four main types of deoxynucleotides in DNA: dAMP, dGMP, dCMP and dTMP. dGMP is used as a substrate for guanosine kinase to form dGDP, which can be phosphorylated to dGTP to support DNA biosynthesis [43]. Emerging evidence demonstrates that nucleotide-free diets could induce immune suppression and affect intestinal function dramatically. The effect of dGMP on the spontaneous proliferation of Caco-2 cells was confirmed [44]. dGMP could stimulate immune cell growth and function, and the influenza virus antigen could be used as an immune stimulator in vitro [45,46]. The structure of Caco-2 cells is similar to the intestinal epithelial cells, which is suitable for the cell model of nutrient absorption and energy transport. The Caco-2 cell is also a kind of human colon cancer cell, showing a corresponding reaction mechanism to resist exogenous injury under oxidative damage and other stress conditions. Many behaviors of cancer cells, including over-proliferation, ROS homeostasis regulation and immune evasion, rely largely on augmented nucleotide metabolism. Indeed, deoxyuridine matters have emerged as powerful relievers of oxidative stress [47]. However, the purine deoxy-nucleosides deoxyadenosine and deoxyguanosine can be phosphorylated by deoxycytidine kinase (DCK) to form dAMP and dGMP, which is considered the purine nucleoside–nucleobase salvage pathway [48]. This was also confirmed by changes in 2′-Deoxyguanosine 5′-monophosphate, uridine 5′-monophosphate and inosine involved in purine and pyrimidine metabolism in this study. This might be due to the Nrf2/HO-1 pathway of Caco-2 cells being activated via H_2_O_2_-induced oxidative damage, while the FSVH supplementation would activate the self-protection immune mechanisms of Caco-2 cells, and the differential metabolites of dGMP accumulated in FSVH group further confirmed the enhanced antioxidant capacity of Caco-2 cells (Figure 10).

Metabolomics analysis verified the cellular defense mechanism against oxidative damage. The glutathione metabolism was the enriched metabolic pathway after FSVH intervention. Glutathione is an antioxidant and antidote, which is the first line of defense against oxidative stress [49]. Glutathione metabolism consists of four steps: synthesis, decomposition, transport and loss. Glutathione peroxidase (GPX) is a key enzyme in the oxidative metabolism of GSH. GPX is one of the important free radical trapping enzymes in the body, which oxidizes GSH to GSSG while reducing H_2_O_2_ to H_2_O, blocking the peroxidation of ROS [50]. Nrf2 is the master regulator of antioxidant and cytoprotective genes, which plays a key protective role in cells by prompting glutathione synthesis and ROS scavenging [51]. The increased activity of glutathione metabolism developed as a result of activation of Nrf2 against the background of a high intracellular concentration of ROS [52]. In addition to the increased mitochondrial membrane potential, ROS and MDA reduction and Nrf2/HO-1 pathway activation detected in this study, combined with the enriched glutathione metabolic pathway, there was a regulatory and inhibitory effect on ferroptosis. The antioxidation effect of FSVH exhibited a possible therapeutic strategy on Caco-2 cells via the Nrf2/GSH pathway (Figure 10). This indicated that glutathione metabolism would exert an important influence on cellular protection. Therefore, FSVH could improve the activities of Caco-2 cells under oxidative stress, and the major way was to enhance cell viability.

## 5. Conclusions

To conclude, the model of oxidative damage to human colonic adenocarcinoma Caco-2 cells induced with H_2_O_2_ was established successfully, and the applied dose of FSVH was investigated in this study. FSVH could exert a cellular protective effect and reduce oxidative damage of Caco-2 cells by improving cell viability, resuming the normal growth cycle, inhibiting cell apoptosis, decreasing ROS levels, increasing mitochondrial membrane potential and enhancing intracellular antioxidant indexes. Furthermore, the expression levels of genes and proteins related to antioxidation pathways indicated that FSVH could regulate the oxidative damage defense mechanism of cells by activating the Nrf2/HO-1 pathway. Finally, the detection of cell metabolomics confirmed that FSVH would upregulate differential metabolites such as 2′-Deoxyguanosine 5′-monophosphate and antioxidant-related metabolic pathways such as glutathione metabolism, which further provided evidence for the antioxidant mechanism of FSVH. Above all, FSVH supplementation would be an effective and innovative strategy for reducing H_2_O_2_-induced oxidative damage of Caco-2 cells, opening up a new vision for the development of FSVH in antioxidant engineering and food science.

## Figures and Tables

**Table 1:** Primers used in RT-*q*PCR.

Primers	Sequence (5′→3′)	
HO-1	Forward	5′-AACTTTCAGAAGGGCCAGGT-3′
	Reverse	5′-AGACTGGGCTCTCCTTGTTG-3′
NQO-1	Forward	5′-GGGATCCACGGGGACATGAATG-3′
	Reverse	5′-ATTTGAATTCGGGCGTCTGCTG-3′
β-actin	Forward	5′-GGAAATCGTGCGTGACATTA-3′
	Reverse	5′-GGAGCAATGATCTTGATCTTC-3′

**Table 2:** The main components of SVH and FSVH.

Functional Components	SVH	FSVH
Protein and peptide (g/100 g)	37.05 ± 2.89	32.35 ± 3.89
Polysaccharide (g/100 g)	13.85 ± 2.25	19.53 ± 2.56 *
Saponin (mg/kg)	5187.42 ± 339.15	5537.93 ± 323.14
Cordycepin (mg/kg)	0	852.19 ± 83.54 *

Abbreviation: SVH-Sea cucumber viscera enzymatic hydrolysate; FSVH-Fermented sea cucumber viscera protease hydrolysate. Results were mean ± SD for three experiments. * *p* < 0.05.

**Table 3:** The differential metabolites in negative ion model.

Group Name	Up	Down
H_2_O_2_ vs. Normal	18	8
SVH vs. H_2_O_2_	23	13
FSVH vs. H_2_O_2_	13	28

**Table 4:** Fold change of the differential metabolites in each group.

No.	Name	VIP	*p*-Value	FC (H_2_O_2_ vs. Normal)	FC (SVH vs. H_2_O_2_)	FC (FSVH vs. H_2_O_2_)	Pathway
1	2′-Deoxyguanosine 5′-monophosphate	1.24	0.000026	0.00	2441.43	56076.55	Purine metabolism
2	GDP-L-fucose	1.02	0.005034	0.01	121.51	71.05	Fructose and mannose metabolism
3	Catechin	1.15	0.001565	0.03	11.97	3.44	Flavonoid biosynthesis
4	Uridine 5′-monophosphate	1.10	0.011365	0.11	4.27	2.77	Pyrimidine metabolism
5	Methyl acetate	1.11	0.000007	0.18	36.39	0.06	Butanoate metabolism
6	Succinic acid	1.07	0.000053	0.24	22.71	0.13	Citrate cycle (TCA cycle)
7	Alpha-D-Glucose 1,6-bisphosphate	1.52	0.000067	12.72	0.02	0.02	Starch and sucrose metabolism
8	Inosine	1.57	0.003841	9.36	0.30	0.00	Purine metabolism
9	Phenylacetylglycine	1.39	0.007821	2.14	0.24	0.28	Phenylalanine metabolism
10	Bergapten	1.55	0.000607	2.05	0.25	0.17	Biosynthesis of various plant secondary metabolites
11	Theophylline	1.55	0.007128	1.95	0.64	0.22	Caffeine metabolism
12	Dihydroxyacetone phosphate	1.43	0.003620	1.56	0.03	0.13	Glycolysis/Gluconeogenesis
13	Indoleacetic acid	1.30	0.000001	1.51	79.36	2.96	Tryptophan metabolism

Abbreviation: FC—fold change; GDP—guanosine diphosphate; VIP—variable importance in the projection; SVH—sea cucumber viscera enzymatic hydrolysate; FSVH—fermented sea cucumber viscera protease hydrolysate.

## Data Availability

The datasets generated for this study are available on request to the corresponding author.
